# Antioxidative Responses of Duckweed (*Lemna minor* L.) to Phenol and Rhizosphere-Associated Bacterial Strain *Hafnia paralvei* C32-106/3

**DOI:** 10.3390/antiox10111719

**Published:** 2021-10-28

**Authors:** Olga Radulović, Slaviša Stanković, Olja Stanojević, Zoran Vujčić, Biljana Dojnov, Milana Trifunović-Momčilov, Marija Marković

**Affiliations:** 1 Department of Plant Physiology, Institute for Biological Research “Siniša Stanković”, National Institute of the Republic of Serbia, University of Belgrade, 142 Bulevar Despota Stefana, 11060 Belgrade, Serbia; milanag@ibiss.bg.ac.rs (M.T.-M.); marija.nikolic@ibiss.bg.ac.rs (M.M.); 2 Faculty of Biology, University of Belgrade, 16 Studentski Trg, 11000 Belgrade, Serbia; slavisas@bio.bg.ac.rs (S.S.); olja.stanojevic@bio.bg.ac.rs (O.S.); 3 Department of Biochemistry, Faculty of Chemistry, University of Belgrade, 12-16 Studentski Trg, 11000 Belgrade, Serbia; zvujcic@chem.bg.ac.rs; 4 Department of Chemistry, Institute of Chemistry, Technology and Metallurgy, National Institute of the Republic of Serbia, University of Belgrade, 12 Njegoševa, 11000 Belgrade, Serbia; bdojnov@chem.bg.ac.rs

**Keywords:** phenol, bacteria, duckweed, antioxidative, stress

## Abstract

Duckweed (*L. minor*) is a cosmopolitan aquatic plant of simplified morphology and rapid vegetative reproduction. In this study, an *H. paralvei* bacterial strain and its influence on the antioxidative response of the duckweeds to phenol, a recalcitrant environmental pollutant, were investigated. Sterile duckweed cultures were inoculated with *H. paralvei* in vitro and cultivated in the presence or absence of phenol (500 mg L^−1^), in order to investigate bacterial effects on plant oxidative stress during 5 days. Total soluble proteins, guaiacol peroxidase expression, concentration of hydrogen peroxide and malondialdehyde as well as the total ascorbic acid of the plants were monitored. Moreover, bacterial production of indole-3-acetic acid (IAA) was measured in order to investigate *H. paralvei’s* influence on plant growth. In general, the addition of phenol elevated all biochemical parameters in *L. minor* except AsA and total soluble proteins. Phenol as well as bacteria influenced the expression of guaiacol peroxidase. Different isoforms were associated with phenol compared to isoforms expressed in phenol-free medium. Considering that duckweeds showed increased antioxidative parameters in the presence of phenol, it can be assumed that the measured parameters might be involved in the plant’s defense system. *H. paralvei* is an IAA producer and its presence in the rhizosphere of duckweeds decreased the oxidative stress of the plants, which can be taken as evidence that this bacterial strain acts protectively on the plants during phenol exposure.

## 1. Introduction

The common duckweed (*Lemna minor*, L.) is a rapidly reproducing vascular plant of simplified morphology, with remarkable tolerance to various pollutants. These characteristics make *L. minor* an optimal model organism for toxicity testing as well as wastewater treatment [1,2,3]. In agriculture, due to its rapidly increasing low-starch biomass and ability to thrive under very different conditions, duckweeds are used as a cheap source of protein [4]. Industrial growth, especially in developing countries, results in the constant influx of phenol into aquatic ecosystems, where it causes lethal or toxic damage to all living organisms in a wide range of concentrations from as low as 0.26 to 1204.6 mg L^−1^ [5]. Plants very often grow under toxic conditions, resulting in oxidative stress and accumulation of reactive oxygen species (ROS) which are harmful to cells if they exceed the natural defense mechanisms of plants [6,7,8]. The efficiency of the antioxidative response and level of oxidative stress can be monitored in vitro via specific biochemical markers such as hydrogen peroxide, lipid peroxidation and ascorbic acid. *L. minor* and related duckweeds are remarkably tolerant to oxidative stress caused by organic and inorganic pollutants [9,10,11]. Moreover, certain bacterial strains in the rhizosphere can modulate the antioxidative status of the plants and influence plant biomass production [12,13,14]. Many of these strains are plant-growth-promoting bacteria (PGPB) which can activate plant defense responses on every level, including defense-related genes, expression of enzymes and production of non-enzymatic protective factors [14]. The most common features of PGPB are the production of phytohormones (most notably, indole-3-acetic acid, IAA), solubilization of phosphorus, mobilization of iron (siderophores) and activation of the enzyme aminocyclopropane-1-carboxylate (ACC) deaminase [13,14]. In duckweeds, favorable modulation of antioxidative response is reported to be correlated with plant-growth-promoting abilities of bacteria [15,16]. Our previous studies on duckweeds also showed a promotive effect of the natural bacterial community in rhizosphere, most prominently on the plants’ multiplication [17,18,19]. However, information regarding the effect of rhizosphere-associated bacteria on the oxidative stress response of aquatic plants, especially under the conditions of abiotic stress such as phenol pollution, is still lacking [15,16]. Fully understanding bacteria–duckweed interactions from the perspective of oxidative stress parameters may elucidate these complex mechanisms which are responsible for promotion or inhibition of plant growth. 

The aim of this study was to ascertain whether a bacterial strain, *Hafnia paralvei* C32-106/3, MF526939, isolated from the rhizosphere of *L. minor*, affects the plant’s antioxidative response to phenol. The findings from this study could be utilized in agriculture and in wastewater treatment, as a means of increasing the efficiency of antioxidative response and subsequently the efficiency of biomass production of duckweeds and of other plants. To the best of our knowledge, this is the first study analyzing the plant-growth-promoting abilities of *H. paralvei* and its effects on the antioxidative response of the plants exposed to phenol. In this study, some fundamental parameters of oxidative stress were monitored during five days of in vitro cultivation: peroxidases, ascorbic acid, hydrogen peroxide, lipid peroxidation and total proteins. Furthermore, to determine whether the *H. paralvei* bacterial strain possesses plant-growth-promoting activity, analysis of IAA production was also conducted.

## 2. Materials and Methods

### 2.1. Plant Material and Growth Conditions

*L. minor* was collected from a pond in the garden of the Institute for Biological Research “Siniša Stanković” in Belgrade. Plants were washed with tap water for 20 min and then immediately surface sterilized for 5 min in a commercial bleach solution containing 5% (*v*/*v*) sodium hypochlorite. The excess bleach was washed off with sterile distilled water three times. Sterile duckweed cultures (2–4 fronds) were maintained in Murashige and Skoog medium [20], at 24 ± 2 °C (under fluorescent light of 40 μmol m^−2^ s^−1^ with 16 h light/8 h dark photoperiod). The stock culture and plants exposed to phenol (with or without bacteria) were also kept in growth chambers. Nutrition medium was replaced every 7 days. In all experiments we used complete plants (roots + fronds).

### 2.2. Experiments with Phenol

Flat-bottom glass flasks containing 100 mL of MS medium with sterile duckweeds (approx. 150 fronds in each flask) were used as the basic experimental setting. There were four different groups of samples used: flasks containing only phenol-free MS medium and duckweeds; duckweeds inoculated with bacteria in phenol-free MS medium; duckweeds in phenol-supplemented MS medium; duckweeds inoculated with bacteria in phenol-supplemented MS medium. Phenol concentration in medium was 500 mg L^−1^. Bacterial inoculum was prepared from 1 mL of fresh overnight culture grown in Luria–Bertani (LB) medium under shaking conditions (180 rpm). This volume was briefly centrifuged and the supernatant was discarded. The cells were gently washed in MS medium and transferred to flasks containing duckweeds in MS medium with or without phenol, respectively.

### 2.3. Bacterial Strain Hafnia Paralvei Strain C32-106/3 (Accession Number MF526939)

*H. paralvei* was isolated and identified in our previous studies and selected for the ensuing experiments due to its high resistance to phenol, ability to eliminate phenol and a positive effect on the multiplication rates of the duckweeds in vitro [18,19]. Bacterial cultures were grown and maintained on a Luria–Bertani (LB) medium, prepared according to the previously applied protocol [17].

### 2.4. Estimation of H. paralvei Density on the Surface of L. minor 

An overnight culture of *H. paralvei* in 5 mL of LB medium was used to inoculate MS medium with or without phenol (1% *v*/*v*). The amount of bacterial cells on the plant surface was estimated through the number of colony-forming units (CFU) per milligram of fresh weight of plants. After 5 days of co-cultivation, 0.1 g of fresh weight of the plants was collected, vortexed in 1 mL of sterile MS medium and serially diluted. The diluted suspension was then streaked onto solid LB medium containing 1.5% agar. After an overnight incubation, CFU numbers were counted. Results were presented as CFU per milligram of fresh weight (FW) of the plants.

### 2.5. Antioxidative Enzymes

#### 2.5.1. Extraction of Total Proteins

Fronds and roots (1 g) were homogenized in liquid nitrogen with mortar and pestle and 0.1 M potassium phosphate (K-P) extraction buffer (0.1 M ammonium-acetate pH 6.8 pH with 1.5% insoluble polyvinylpyrrolidone (PVPP)), 10 mM dithiothreitol (DTT) and 1 mM phenylmethylsulfonyl fluoride (PMSF) were added. After the homogenization, the samples were centrifuged at 12,000× *g* for 5 min at 4 °C. Isolates were kept at −80 °C and used for protein quantification and enzyme tests. Total proteins were quantified according to Bradford (1976) [21]. For this analysis, a mixture of sample (10 µL) and Bradford reagent (200 µL) was prepared. The absorbances of triplicates were measured spectrophotometrically at 595 nm. Based on the standard curve, made using bovine serum albumin (BSA) solution, total protein concentration was calculated. The results were presented graphically.

#### 2.5.2. Isoelectric Focusing and Zymogram Detection of Guaiacol Peroxidase (GPX, EC 1.11.1.7)

Isoelectric focusing and zymogram detection of GPX were performed according to Vujčić et al. (2015) [22]. For the separation of GPX isoforms and isoelectric focusing, polyacrylamide gel was used, containing 3.75 mL of acrylamide, 4 mL of glycerol, 6.5 mL of deionized water, 12 µL of tetramethylethylenediamide (TEMED) and 75 µL of ammonium persulfate (APS). 

Isoelectric focusing was performed using a Multiphor II electrophoresis system (Pharmacia-LKB Biotechnology, Uppsala, Sweden) according to the manufacturer’s instructions. Focusing was carried out on a gel with ampholytes in a pH range of 3.0–10.0, at 1200 V of constant power for 3 h at 10 °C. A broad pI kit (GE Healthcare) was used for the isoelectric point (pI) markers. 

Zymogram detection of GPX was performed after the gel was washed twice (5 min) with distilled water and then equilibrated with 100 mM phosphate buffer, pH 7.0 twice (each 5 min duration). Thereafter, the gel was dipped into 8.8 mM guaiacol solution in 100 mM phosphate buffer, pH 7.0 with 4.4 mM H_2_O_2_ and incubated at 25 °C for 10 min until the visualization of dark brown color bands indicating POX presence according to the modified method of Siegel and Galston [23]. Gel analysis was performed using the TotalLab TL 120, Newcastle-Upon-Tyne, UK graphics package.

### 2.6. Determination of Hydrogen Peroxide

Content of hydrogen peroxide (H_2_O_2_) was measured according to Sergiev et al. (1997) [24]. Plant material (0.1 g) was finely ground in liquid nitrogen, and the extraction was performed with 3.75 mL of 0.1% trichloroacetic acid (TCA). The homogenate was centrifuged (15000× *g*, 4 °C, 15 min) and the supernatant was further mixed with 10 mM potassium phosphate buffer (pH 7.0) and 1 M potassium iodide (1:3:2). Absorbances were read at 320 nm. The concentration of H_2_O_2_ was calculated according to the standard curve with known concentrations of H_2_O_2_ and presented graphically, as mM of H_2_O_2_ g^−1^ FW.

### 2.7. Lipid Peroxidation (MDA Content)

Lipid peroxidation was measured as malondialdehyde (MDA) content according to Velikova et al. (2000), with some modifications [25]. The plant material (0.1 g) was ground with an ice-cold mortar and pestle and homogenized in 1 mL of 0.1% TCA. The homogenate was centrifuged at 15000× *g*, 4 °C for 10 min. The supernatant was mixed with 20% TCA and 0.5% 2-thiobarbituric acid (TBA) 1:3 (w:w) in water bath at 95 °C for 30 min and then cooled on ice. After centrifugation at 4 °C for 10 min, MDA was determined spectrophotometrically. Absorbances of supernatant were read at 520 nm and 600 nm. The concentration of MDA was calculated according to the extinction coefficient (155 mM^−1^cm^−1^) of the red complex formed between MDA and TBA and presented graphically, as µM of MDA per gram of FW of the plants.

### 2.8. Determination of Ascorbic Acid

Ascorbic acid was estimated according to Mukherjee and Choudhuri (1983) [26]. Briefly, plant material (0.1 g fresh weight) was frozen in liquid nitrogen and immediately finely ground with pestle and mortar. Extraction was performed in 2 mL of extracting solution (5% metaphosphoric acid dissolved in 10% glacial acetic acid). The extract was mixed with dinitrophenylhydrazine (2%) and 10% thiourea, incubated in boiling water and quickly cooled afterward using an ice bath. The reaction was stopped by adding 85% sulfuric acid, also on ice. The absorbance was read at 530 nm. The concentration of ascorbic acid was calculated from the standard curve with known concentrations of ascorbic acid. 

### 2.9. Analysis of IAA Production

Quantification of IAA was performed according to Gordon and Weber (1951) [27]. Briefly, 100 mL of LB medium was supplemented with 0.1% tryptophan in a flat-bottomed glass flask (1000 mL). One half of the samples were supplemented with phenol (500 mg L^−1^) while the other half was phenol-free. The final pH values of the media were 5.5, 7.0 and 8.5 in order to examine the influence of different pH. All samples were inoculated with one loop full of bacterial stock, with the exception of the negative control where sterile medium was used. The media were incubated for 2 days at room temperature, under shaking conditions (100 rpm). Medium was sampled (1 mL), centrifuged (10,000× *g*) and the supernatant mixed with 2 mL of Salkowski reagent (0.5 M ferric chloride solution in 70% perchloric acid, diluted with distilled water). Samples were incubated in the dark for 30 min, at room temperature. The development of red color was indicative of IAA formation. The absorbance was read at 540 nm. The concentration of IAA was calculated from the standard curve with known concentrations of IAA. IAA content was expressed in µg of IAA per mg of bacterial biomass.

### 2.10. Statistical Analysis

Each experiment was repeated independently at least three times. The number of replicates is indicated in the caption of each figure. Numerical data were analyzed using the computer program GraphPad Prism 9.1.0, San Diego, CA, USA. Each value represents the mean ± standard error (SE). The significance of difference of treatments was tested by Student’s paired t-test. The difference was considered significant at *p* less than 0.05 (*p* < 0.05).

## 3. Results

### 3.1. Bacterial Density on the Duckweeds’ Surface

**Figure 1:**
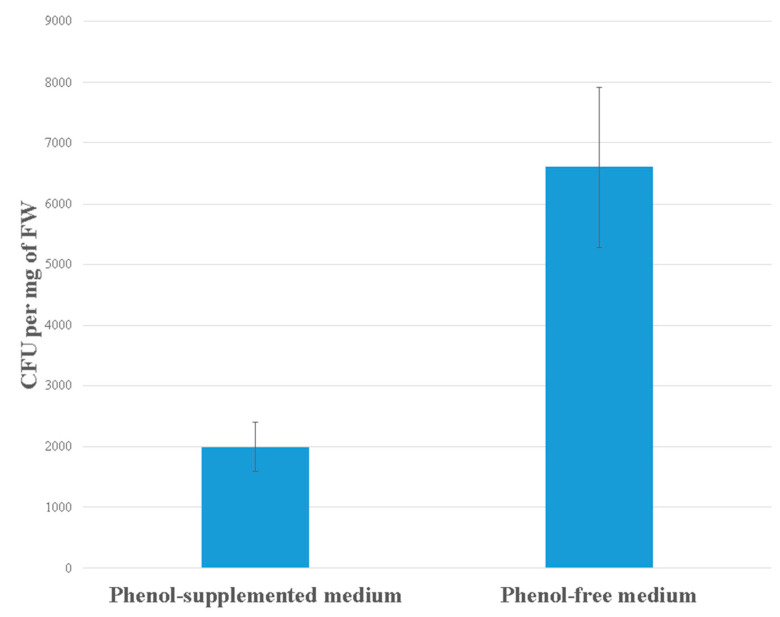
Estimated numbers of colony-forming units (CFU) on the plant surface of the duckweeds presented as number of colonies (CFU) per one milligram of fresh weight of intact duckweeds in MS medium with phenol (500 mg L^−1^) or without phenol after 5 days of co-cultivation of duckweeds with bacteria (*H. paralvei*). Data represent the mean ± SE (*n* = 3).

Bacterial density on the plant surface was relatively low, despite the dense inoculum in the free medium (Figure 1). After 5 days of co-cultivation with the duckweeds, there were approximately 2000 CFU per 1 mg of fresh weight (FW) of duckweed in phenol-supplemented medium. Bacterial density was increased more than threefold in phenol-free medium (6600 ± 1300 CFU per 1 mg FW). Since an average bacterial cell weighs an amount of approximately 1 pg, the total biomass of bacteria on the surface of duckweeds was (2 ± 0.4) ng and (6.6 ± 1.3) ng, respectively.

### 3.2. Total Soluble Proteins

**Figure 2:**
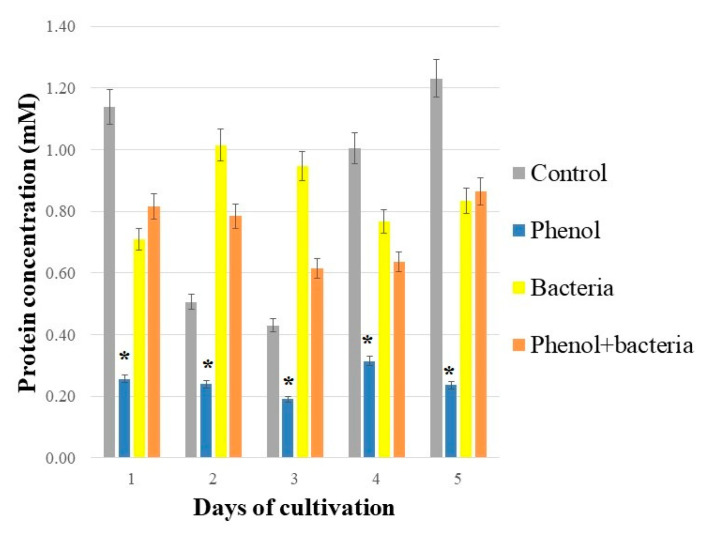
Total soluble proteins of duckweeds cultivated under different experimental conditions: Control—sterile duckweeds cultivated in phenol-free MS medium; Phenol—sterile duckweeds cultivated in phenol-supplemented MS medium; Bacteria—duckweeds inoculated with bacteria *H. paralvei* and cultivated in phenol-free MS medium; Phenol + bacteria—duckweeds inoculated with bacteria *H. paralvei* and cultivated in phenol-supplemented MS medium. Concentration of phenol in all experiments was 500 mg L^−1^. Data represent the mean ± SE (*n* = 3). * indicates significant difference in treatments at *p* < 0.05.

Phenol decreased the concentration of total soluble proteins in duckweeds compared to duckweeds cultivated in phenol-free MS medium (Figure 2). The same trend was noticed when the plants were inoculated with bacteria (*H. paralvei*). Bacteria on plant surface had a positive effect on total soluble protein content in both phenol-free and phenol-containing MS media. A statistically significant difference was observed between Control and all other treatments (Figure 2). No statistically significant difference was found between Control and Bacteria; Control and Phenol + bacteria; Bacteria and Phenol + bacteria. On average, protein content in Phenol (duckweeds cultivated in sterile phenol-supplemented MS medium) showed a 71.25% decrease compared to Control (duckweeds cultivated in sterile phenol-free MS medium). 

### 3.3. Zymogram Detection of GPX

**Figure 3:**
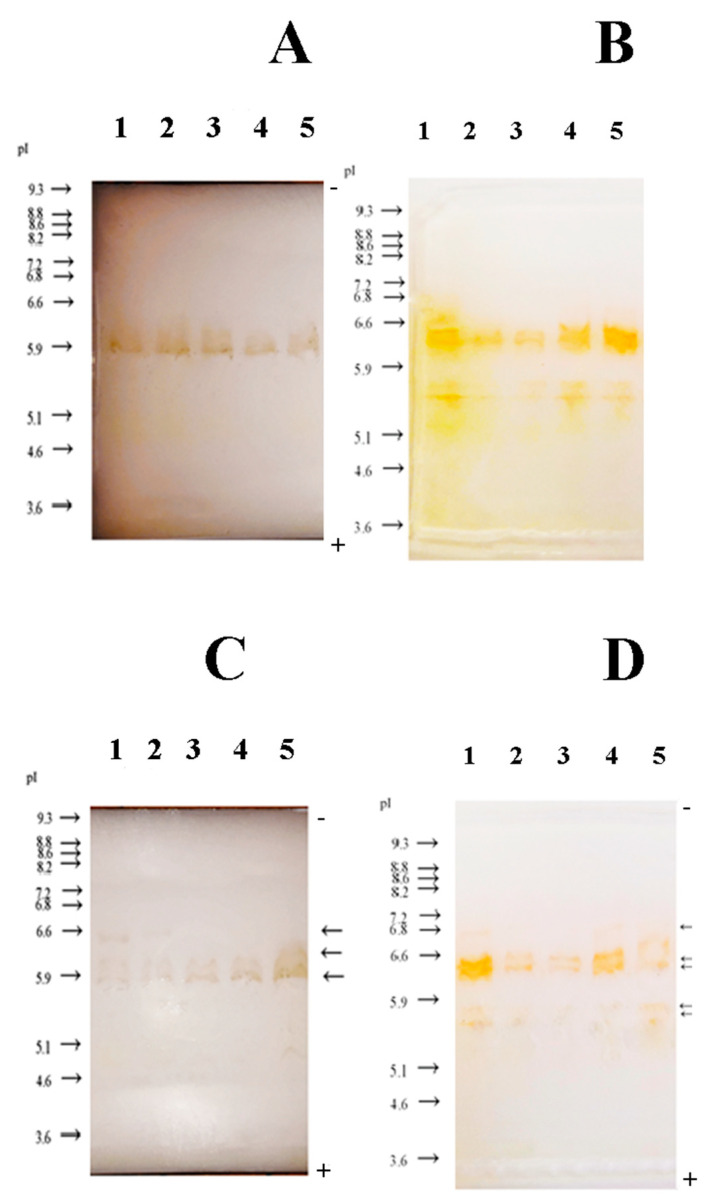
Zymogram detection of guaiacol peroxidase (GPX): (**A**) sterile duckweeds cultivated in phenol-free MS medium (control plants); (**B**) sterile duckweeds cultivated in phenol-supplemented MS medium; (**C**) duckweeds inoculated with bacteria *H. paralvei* and cultivated in phenol-free MS medium; (**D**) duckweeds inoculated with bacteria *H. paralvei* and cultivated in phenol-supplemented MS medium. Numbers (1–5) represent days of cultivation. Concentration of phenol in all experiments was 500 mg L^−1^. pI—isoelectric point.

Three different isoforms of GPX were detected in phenol-free medium, all of which were detected in plants cultivated with bacteria (Figure 3A and 3C. All detected isoforms appeared in the acidic region at different pI values (5.9 and 6.6) compared to GPX detected in phenol-supplemented medium. During the first 4 days of co-cultivation, the intensity of bands corresponding to different isoforms was diminished (Figure 3C). Only one isoform was detected in sterile duckweeds, at pI around 5.9. The intensity of this single band did not change over time (Figure 3A).

Five different isoforms of GPX with different pI values (one at 6.7, two at 6.5 and two at 5.5, respectively) were detected in phenol-supplemented medium (Figure 3B and 3D). All detected isoforms were in the acidic region. After five days of co-cultivation with bacteria, a decrease in the intensity of bands corresponding to different isoforms was observed (Figure 3D).

### 3.4. Hydrogen Peroxide (H_2_O_2_) 

**Figure 4:**
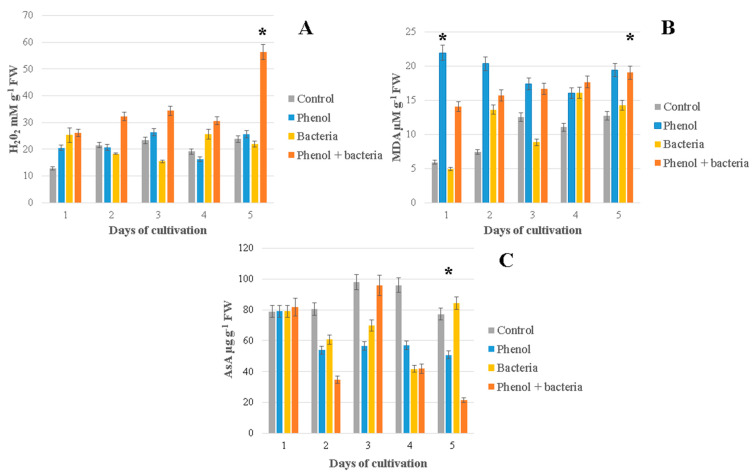
Non-enzymatic antioxidative parameters of duckweeds cultivated under different experimental conditions: (**A**) hydrogen peroxide (H_2_O_2_); (**B**) malondialdehyde (MDA); (**C**) ascorbic acid (AsA). Control—sterile duckweeds cultivated in phenol-free MS medium; Phenol—sterile duckweeds cultivated in phenol-supplemented MS medium; Bacteria—duckweeds inoculated with bacteria *H. paralvei* and cultivated in phenol-free MS medium; Phenol + bacteria—duckweeds inoculated with bacteria *H. paralvei* and cultivated in phenol-supplemented MS medium. Concentration of phenol in all experiments was 500 mg L^−1^. Data represent the mean ± SE (*n* = 3). * indicates significant differences between treatments at *p* < 0.05.

Hydrogen peroxide concentration is significantly higher in duckweeds co-cultivated with bacteria in phenol-supplemented MS medium compared to the sterile duckweeds grown in phenol-supplemented MS medium (Figure 4A). In all other instances, there was no statistically significant difference between treatments. The maximum amount of hydrogen peroxide was achieved after 5 days of cultivation in plants grown with *H. paralvei* and phenol. On the last day of co-cultivation of duckweeds and bacteria in phenol-supplemented medium, the amount of hydrogen peroxide was almost two times higher compared to previous days (approx. 56.296 mM g^−1^ FW after day 5 compared to approximately 30 mM g^−1^ FW on days 1–4). 

### 3.5. Lipid Peroxidation (MDA Content)

Phenol had a visible effect on the lipid peroxidation and consequently MDA content in plant tissue, throughout the experiment. The highest level of MDA was measured after one day in sterile plants cultivated in phenol-containing medium. During treatment, MDA content decreased constantly in this group of duckweeds, whereas it increased almost linearly in all other specimens, including sterile duckweeds cultivated in phenol-free medium. MDA levels in duckweeds cultivated without phenol were lower than MDA levels in duckweeds cultivated with phenol. After five days, MDA concentration was almost the same in plants inoculated with bacteria both in phenol-free and in phenol-containing medium. However, statistical analysis of different treatments showed that there is no statistically significant difference in the total amount of MDA between the samples with sterile duckweeds cultivated in phenol and samples with bacteria and phenol or between the samples with sterile duckweeds and duckweeds inoculated with bacteria (Figure 4B). There was a significant difference in MDA content of sterile duckweeds cultivated in phenol-free medium and duckweeds cultivated in phenol-containing medium. Likewise, there was significant difference between samples with sterile duckweeds in phenol-free medium and duckweeds cultivated in phenol-containing medium with bacteria. There was no difference between samples of duckweeds inoculated with bacteria and samples of sterile duckweeds cultivated in phenol-supplemented medium. 

### 3.6. Ascorbic Acid

A constantly high level of ascorbic acid was detected in *L. minor* cultivated with bacteria under phenol-free conditions. After five days, AsA concentration was almost the same in duckweeds cultivated in phenol-free medium, regardless if they were co-cultivated with bacteria. Likewise, after five days, AsA concentration was equal in duckweeds cultivated in phenol-supplemented media, regardless of whether they were co-cultivated with bacteria; this concentration was higher than in phenol-free conditions (Figure 4C). AsA was significantly lower only in sterile duckweeds cultivated in phenol-supplemented medium compared with sterile duckweeds cultivated in phenol-free conditions. 

### 3.7. IAA Production

**Figure 5:**
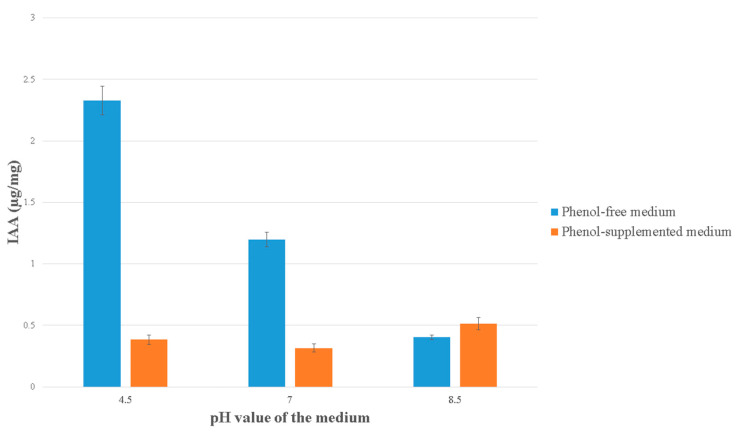
Bacterial indole-3-acetic acid (IAA) production in LB medium with different pH values: phenol-supplemented medium—LB medium with added phenol (500 mg L^−1^ final concentration); phenol-free medium—LB medium without phenol. Data represent the mean ± SE (*n* = 3).

The highest amount of IAA was produced in acidic phenol-free LB medium, at pH 4.5 (Figure 5). Half of that amount was produced in phenol-free LB medium at pH 7.0, and the least amount was produced in phenol-free LB medium at pH 8.0. The amount of IAA detected in phenol-supplemented LB media was consistently low, with the greatest difference at pH 4.5 and 7 compared to phenol-free LB medium. 

## 4. Discussion

Rhizospheric bacteria exert various effects on the plants. Their most obvious effect, i.e., the ability to remove different toxic compounds and in this case, to remove phenol, is well-researched [28,29,30]. Their ability to promote growth of plants was investigated in great detail, almost exclusively in the light of crop production and plants’ immunity [14]. However, the link between bacterial growth promotion and oxidative stress in plants is still poorly understood, especially in aquatic plants [15]. Even less is known about the role of bacteria in alleviating oxidative stress of plants in the context of phytoremediation. Therefore, in this study, we used a bacterial strain with reported positive effects on the multiplication rate of duckweeds, *H. paralvei* [19], and analyzed its effects on the antioxidative parameters of duckweeds. 

*H. paralvei* is an actively motile bacterium which was only partially found on the surface of duckweeds [19]. Despite the low density of bacterial colonies on the surface of duckweeds, their presence significantly affected some aspects of *L. minor* antioxidative response. The presence of *H. paralvei* colonies also increased the amount of total proteins in duckweeds, which can be described as a protective effect of bacterial presence on plant surface. Since bacterial biomass on duckweeds’ surface was very low, this total protein increase was not a result of passive addition of bacterial proteins. 

Peroxidases (POX, EC 1.11.1.7) are ubiquitous enzymes in the plants [31]. Those enzymes are activated as a response to various noxious events, including inorganic and organic pollutants. Oxidation of phenol and its derivatives is one of the main functions of POXs [32].

Guaiacol-peroxidase (GPX) had different and distinct isoforms depending on whether duckweeds were cultivated in a phenol-supplemented medium or in a phenol-free medium with *H. paralvei*. This is in correlation with existing literature, where it was reported that there were specific stress-related isoforms of GPX [33]. Bacteria also led to a decrease in GPX expression in phenol-supplemented and in phenol-free medium in a time-specific manner. All isoforms of GPX showed lower intensity at the end of treatment during cultivation with phenol and bacteria. The increase in expression of GPX after 24 h of exposure to phenol indicates that *L. minor* was in a state of oxidative stress. Similar results were reported by Ibanez et al. [34] where the activity of peroxidases of *Vicia sativa* increased after 7 days of treatment and was dose-dependent: the increase in activity was not observed at concentrations of phenol lower than 500 mg L^−1^. A similar increase in GPX activity was detected when *L. minor* was subjected to copper ions (Cu^2+^) [35]. Increase in GPX activity indicates the importance of rapid response of GPX to stress. However, according to Basiglini et al. [3], a complex wastewater mixture containing high concentrations of various organic and inorganic pollutants caused a decline in the activity of guaiacol peroxidases, ascorbate peroxidases and catalases in duckweeds. This underlines the significance of the type and complexity of stressors that duckweeds are exposed to.

When phenol was not added to the medium, bacteria apparently induced the expression of three distinct isoforms of GPX that were different from those expressed in the presence of phenol, showing that the GPX expression was indeed stressor-specific and dependent on whether the stressor was abiotic (phenol) or biotic (bacteria). Similarly, Ishizawa et al. [15] reported that PGPB strains in their study increased the activity of GPX, whereas the inhibitory strains decreased its activity. 

It is possible that PGPB acts protectively only under certain conditions, which was observed in some studies [16,29,36,37]. If the plants are cultivated under neutral or positive conditions, the antioxidant activity of PGPB might not be activated, or their PGPB activity might depend on mechanisms other than antioxidative protection, as observed by Ishizawa et al. [15,16]. The interactions between bacteria and plants are dynamic and dependent on other external conditions as well. Different studies report the differences in oxidative stress response depending on the duration of exposure to abiotic stressor and the type of abiotic stressor—while exposure to copper and zinc will boost the antioxidant activity of a PGPB, poor nutrient conditions and hypersalinity will inhibit it [9,15,16].

In addition to their direct positive effect on plants’ growth, some bacteria are also able to actively remove and metabolize the toxic compound that is causing oxidative damage on the plants [28,29,30]. In our previous study, we established that *H. paralvei* removes phenol in synergy with duckweeds [19]. Therefore, it can be assumed that at least some of the positive effects of *H. paralvei* on duckweeds cultivated in phenol-supplemented MS medium can be attributed to their active removal of phenol from the environment.

Total soluble proteins of the plants are a good, simple indicator of plants’ stress [8]. As a general rule, oxidative stress will cause a decrease in the amount of soluble proteins by damaging cellular organelles and inhibiting de novo synthesis of the proteins [9]. It is well-established that proteins are particularly susceptible to phenol toxicity—this is the main reason why phenol is so toxic to all living organisms [5]. We also observed a considerable decrease of total soluble proteins (more than 70%) in duckweeds exposed to phenol. However, when the plants were co-cultivated with *H. paralvei* in phenol-supplemented MS medium, the amount of soluble proteins was comparable to that of control plants. In our control group, we observed a fluctuation in the amount of soluble proteins in untreated plants (cultivated in phenol-free, sterile MS medium). This fluctuation can be attributed to the moment in the growth cycle of duckweeds when they are not as metabolically active due to the limited amount of nutrients in the medium. On average, as expected, the amount of total soluble proteins was the highest in the untreated group of duckweeds compared to the treated groups. The fact that the addition of bacteria to a phenol-supplemented MS medium led to only a slight decrease in the amount of soluble proteins (as opposed to phenol alone, which caused a considerable decrease in the amount of total soluble proteins) suggests that bacteria have a protective effect on the duckweeds. This, together with the fact that bacteria led to a relative decrease in total soluble proteins in duckweeds cultivated in phenol-free MS medium, might suggest that the protective effect of *H. paralvei* depends on the context: namely, that *H. paralvei* will exert some positive effects on the plants only under stressful conditions. 

*H. paralvei* did not significantly increase H_2_O_2_ production in plants cultivated in phenol-free medium. Only in the presence of phenol and bacteria combined is there a significant increase of H_2_O_2_ production in duckweeds. This significant increase can be explained as activation of plants’ defense mechanisms against phenol through the accumulation of H_2_O_2_. Although H_2_O_2_ is toxic to plants if accumulated, it is an essential part of the bio-Fenton reaction in which phenol is degraded in the presence of H_2_O_2_, ferrous ions and peroxidases [38]. Since the bacterial biomass on the surface of the duckweeds was low, this increase in H_2_O_2_ production cannot be attributed to bacteria. Moreover, even non-pathogenic bacteria may cause a certain level of oxidative damage to the plant [39]. The accumulation of hydrogen peroxide in duckweeds in the response to the bacterial presence, as reported by Ishizawa et al. [15] will not diminish the plant-growth-promoting effects of the bacteria. What is important is that there is a favorable cost/benefit ratio and that the oxidative damage caused by bacteria is not overwhelming. That could be a possible explanation for high H_2_O_2_ production in the presence of phenol after five days. GPX showed the highest level of expression at the same time (after five days) which was in accordance with hydrogen peroxide accumulation. An increasing amount of H_2_O_2_ could be very useful in oxidizing phenol even if it enters the cells of plants or bacteria. One of the usual responses to bacterial colonization of plants is spontaneous generation of O_2_^−^ by plants, which did not occur in the aseptic control plants [39,40,41]. This was opposite to our results where bacterial inoculation seemed to have a calming effect on oxidative stress. 

The amount of MDA was significantly increased in the presence of phenol compared to phenol-free conditions, which was an expected negative effect of phenol on the cell walls and membranes. The ability of plants to keep the MDA level constant when exposed to phenol indicated plants’ tolerance of phenol, as was shown in experiments with phenol-tolerant *Vicia sativa* [34]. Similar conclusions were drawn in our previous studies, where we reported that the duckweeds continued reproducing even at high concentrations of phenol and were even able to remove it from their environment [17,19]. Duckweeds’ high tolerance of phenol and ability to remove it were reported in many other studies as well [42,43]. 

*H. paralvei* apparently diminished the MDA content in duckweeds exposed to phenol. Although the presence of bacteria led to an increase in MDA content, this increase was less pronounced than the increase caused by phenol. Duckweeds exposed to phenol and cultivated with bacteria had less MDA than sterile duckweeds cultivated in phenol-supplemented medium, especially in the beginning of co-cultivation (first 3 days). Therefore, by decreasing MDA production in duckweeds exposed to phenol, it can be concluded that *H. paralvei* has at least a mildly protective, antioxidant effect on the membranes of the plants, and this is despite the fact that bacterial density on the plants’ surface was low. 

In the presence of phenol and bacteria, both H_2_O_2_ and MDA were increased after 5 days in contrast to GPX whose expression was decreased. A possible explanation could be that GPX was activated at the beginning of the stress response and then partially inhibited by bacteria by the end of the fifth day of co-cultivation. At the same time, H_2_O_2_ and MDA accumulated in the cells and needed more time to be removed by the plant. The diminished expression of GPX after five days of co-cultivation in phenol could be the cause of the increase in total H_2_O_2_ as GPX oxidizes phenol at the expense of H_2_O_2_ [44]. PGPB in general tends to decrease the activity of GPX, while bacteria with an inhibitory effect on plant growth increase its activity and therefore increase oxidative stress [15]. The decrease in GPX expression might have an antioxidant, protective effect on the duckweeds exposed to phenol. 

It was reported that abiotic stress (e.g., light, wind, salinity) and pollution (e.g., bisphenol A) increase AsA production in some plants [45]. Others, however, respond to abiotic stress (drought) with a reduction in AsA synthesis and, subsequently, in total AsA content [8]. Sterile duckweeds in phenol-supplemented MS medium have significantly lower AsA levels than plants grown in phenol-free conditions. Consistently low levels of AsA in phenol-supplemented medium probably reflect the loss of AsA due to the well-researched phenol-induced damage of mitochondria and chloroplasts [46,47]. After five days of co-cultivation with bacteria in phenol-supplemented medium, AsA content was very low. However, AsA content fluctuated in these specimens and reached its peak after three days of co-cultivation when its level was almost equal to the level observed in phenol-free medium with bacteria. Total amount of AsA was the highest in duckweeds cultivated with bacteria in phenol-free medium. In a similar study with duckweeds and PGPB, ascorbate peroxidases, antioxidative enzymes that deplete AsA, showed diminished activity [15]. This might explain, at least partially, the increase in AsA content in duckweeds co-cultivated with bacteria. It is possible that there is a more complex crosstalk between the bacteria and the AsA de novo synthesis/depletion in the presence of phenol. 

In our previous work, we observed a significant positive effect of *H. paralvei* on the multiplication rates of the duckweeds [19]. It was unclear whether plants multiplied due to higher nutrient content originating from dead bacterial cells, or potentially from bacterial plant-growth-promoting factors (e.g., IAA). In this study, the production of IAA by *H. paralvei* strain in phenol-free medium was dependent on the acidity of the medium, which is in line with existing literature [48]. The acidic environment was correlated with an increase in the amount of IAA, while neutral and basic solutions had an inhibitory effect. Even in the presence of phenol, bacteria were still metabolically active and still produced IAA which is a known PGPB factor. For the purposes of IAA detection, rich nutrient medium (LB) was used. In the natural environment, decaying biological material from plants and other organisms is the source of tryptophan that will be converted into IAA by bacteria. The number of free-living bacteria as well as the amount of naturally occurring tryptophan is therefore low; but long-term coexistence of duckweeds and bacteria ensures a constant PGPB stimulus by IAA-producing bacteria. 

However, the effects of IAA on duckweed growth are still poorly understood. Idris et al. [49] used different mutants of plant-growth-promoting *Bacillus amyloliquefaciens* FZB42 with impaired IAA production that was correlated with reduction in growth promotion of *L. minor,* suggesting that IAA is a plant-growth-promoting factor for duckweeds. 

Another study found that exogenous IAA, dissolved in nutrient solution, had no apparent positive effect on the duckweeds’ growth (multiplication rates and biomass production) at all concentrations tested [50]. However, the same research group reported significant shortening of the roots due to IAA in the medium, suggesting that IAA had at least some positive effect on duckweeds. It is known that roots of duckweeds will elongate under unfavorable conditions (e.g., lack of nutrients) and will shorten under favorable conditions [1]. It is possible that the source of IAA (bacteria or exogenous IAA dissolved in medium) determined the outcomes. Unlike terrestrial plants, the effects of growth regulators on biomass production of aquatic plants have rarely been investigated and the available research is even contradictory [50]. Further investigation is therefore necessary. 

## 5. Conclusions

To the best of our knowledge, this is the first study to analyze different effects of *H. paralvei* on the oxidative stress of *L. minor* in the presence or absence of phenol. The levels of non-enzymatic oxidative stress parameters (H_2_O_2_, MDA and AsA) as well as of total soluble proteins suggest that duckweeds are resistant to phenol and that the presence of bacteria is associated with lower oxidative stress. Compared to phenol, bacteria had a less negative effect. Bacteria regenerated total AsA content in phenol-supplemented medium and modulated the expression of GPX which can decrease oxidative stress, especially in the presence of phenol. Our findings support the hypothesis that the antioxidative response is specific to different types of stressors and to different species of plants. Furthermore, this bacterial strain is an IAA producer and has a positive effect on the multiplication rates of the duckweeds as already shown in our previous work. Since high concentrations of phenol will inhibit the growth of plants due, in part, to oxidative stress, this strain might be employed to promote the growth of plants indirectly, by enhancing their antioxidative response, and directly, through IAA production. We propose that the positive effects of *H. paralvei* on the plants’ oxidative stress are potentially very significant for the removal of environmental contaminants and that the bacteria/duckweeds interactions in the context of antioxidative responses should be the focus of future research.

## Data Availability

The data presented in this study are available in the article.
